# Development of HEK293T-produced recombinant receptor-Fc proteins as potential candidates against canine distemper virus

**DOI:** 10.3389/fvets.2023.1180673

**Published:** 2023-05-05

**Authors:** Lingling Song, Hu Shan, Juan Huang

**Affiliations:** ^1^College of Veterinary Medicine, Qingdao Agricultural University, Qingdao, China; ^2^Shandong Collaborative Innovation Center for Development of Veterinary Pharmaceuticals, Qingdao, China; ^3^Qingdao Research Center for Veterinary Biological Engineering and Technology, Qingdao, China

**Keywords:** antiviral biological agent, canine distemper virus, Fc-fusion protein, Nectin-4, SLAM

## Abstract

Canine distemper (CD) is a highly contagious viral disease worldwide. Although live attenuated vaccine is available as a preventive measure against the disease, cases of vaccination failure highlight the importance of potential alternative agent against canine distemper virus (CDV). CDV infects cells mainly by binding signaling lymphocyte activation molecule (SLAM) and Nectin-4 receptor. Here, to develop a new and safe antiviral biological agent for CD, we constructed and expressed CDV receptor proteins fused with Fc region of canine IgG-B, namely, SLAM-Fc, Nectin-Fc and SLAM-Nectin-Fc in HEK293T cells, and antiviral activity of these receptor-Fc proteins was subsequently evaluated. The results showed that the receptor-Fc proteins efficiently bound to receptor binding domain (RBD) of CDV-H, meanwhile, these receptor-Fc proteins competitively inhibited the binding of His-tagged receptor proteins (SLAM-His or Nectin-His) to CDV-H-RBD-Flag protein. Importantly, receptor-Fc proteins exhibited potent anti-CDV activity *in vitro*. Treatment with receptor-Fc proteins at the pre-entry stage dramatically suppressed CDV infectivity in Vero cells stably expressing canine SLAM. The minimum effective concentration (MEC) of SLAM-Fc, Nectin-Fc and SLAM-Nectin-Fc was 0.2 μg/mL, 0.2 μg/mL, 0.02 μg/mL. The 50% inhibition concentration (IC_50_) of three proteins was 0.58 μg/mL, 0.32 μg/mL and 0.18 μg/mL, respectively. Moreover, treatment with receptor-Fc proteins post viral infection can also inhibit CDV reproduction, the MEC of SLAM-Fc, Nectin-Fc and SLAM-Nectin-Fc was same as pre-treatment, and the IC_50_ of receptor-Fc proteins was 1.10 μg/mL, 0.99 μg/mL and 0.32 μg/mL, respectively. The results suggested that the receptor-Fc proteins were more effective for pre-entry treatment than post-infection treatment, furthermore, SLAM-Nectin-Fc was more effective than SLAM-Fc and Nectin-Fc. These findings revealed the receptor-Fc proteins were promising candidates as inhibitor against CDV.

## Introduction

Canine distemper virus (CDV) is a highly fatal virus that causes severe diseases in many hosts. It not only harms domestic dogs and fur animals, but also threatens the health of wild animals, especially rare and endangered animals such as giant panda and African wild dog (1). CDV spreads across hosts and has caused serious threat in non-human primates (2, 3), and CDV was supposed to be the possible etiology of human multiple sclerosis (4), which raises the concern about possibility of zoonosis. Live attenuated vaccine is widely used for CDV control strategy in most countries. This strategy is important in controlling this virus in dogs, however, maternal antibodies can affect the immune effect of the canine, and the occurrence of canine distemper (CD) in immunized animals has been reported (5–7). Moreover, there is evidence of vaccine-induced CDV infections in wildlife, for example, CDV cases in wildlife due to the vaccine strain infection have been reported in South Africa (8). Therefore, there is an urgent need to develop novel effective treatment methods.

CDV is a member of the genus *Morbillivirus*, family *Paramyxoviridae*, which is a single-stranded non-segmented negative sense RNA virus (9). The genome encodes 6 structural proteins: nucleoprotein (N), phosphoprotein (P), matrix protein (M), fusion protein (F), haemagglutinin (H) and large protein (L) (10). The viral ribonucleoprotein (RNP) complex comprises the RNA genome combined with N, L and P protein. M protein located between the nucleocapsid and the envelope is responsible for the transcription and budding of the virus (11). The H and F proteins are viral envelop glycoproteins that involve in the virus-receptor recognition and host cell entry, especially the H protein is the key determinant in viral entry by mediating virus-receptor binding and initiating viral infection (10). The ectodomain (aa 59 to 604 or 607) of H protein comprises three main domains: stalk, neck, and C-terminal head, the head domain is the major receptor-binding domain (RBD) (12–14). The H gene sequence is highly variable, with 19 genotypes currently reported (15), the variability and genetic diversity of CDV limits the virus-targeted control strategy of CD.

Currently, two types of host cell receptors have been identified that play a key role in the pathogenesis of CDV. CDV establishes the infection by binding signaling lymphocyte activation molecule (SLAM) receptor expressed by dendritic cells, subsets of thymocytes, macrophages, and T- and B- lymphocytes, so, the SLAM receptor is associated with immune-suppression (16). CDV initiates viral infection by binding H protein to the V domain of SLAM (17). This facilitates the systemic dissemination of virus interacts with a second receptor: nectin cell adhesion molecule 4 (Nectin-4), expressed at the basolateral surface of epithelial cells (17, 18). CDV-H protein binds closely to the V region of the Nectin-4 receptor and helps the virus attach and invade epithelial cells (19). Finally, the virus invades the central nervous system (CNS), inducing severe neurological disease by establishing persistent infection (16). Considering the important role of SLAM and Nectin-4 in the host recognition of CDV, pathogenicity, and virus transmission (20), SLAM and Nectin-4 receptor may be targets for development of antiviral agents to block CDV infection.

Since the first description in 1989 of CD4-Fc-fusion antagonists that inhibit human immune deficiency virus entry into T cells, Fc-fusion proteins have been intensely investigated for their effectiveness to curb a range of pathologies. In the treatment of human diseases, Fc-fusion strategies have been used in therapy including experimental and clinical drugs (21–23). Fusion proteins based on the immunoglobulin Fc domain show the ability to facilitate protein expression and enable easy purification of recombinant protein by protein A chromatography (24). Additionally, the Fc domain can also prolong the half-life of the proteins. ACE2 is the *in vivo* SARS-CoV-2 functional receptor, recombinant ACE2 (rACE2) was reported to have a short half-life and fast clearance rate in contrast to a rACE2 with an Fc-fusion protein (rACE2-Fc) (25). Meanwhile, it has been shown that ACE2-Fc fusion proteins expressed in both plant and mammalian systems can inhibit SARS-CoV-2 replication (26–28).

In our study, we engineered SLAM and Nectin-4 by fusing the Fc region of canine immunoglobulin IgG-B and transiently expressed the constructs in HEK293T cells using eukaryotic expression vector. *In vitro* antiviral assay, SLAM-Fc, Nectin-Fc and SLAM-Nectin-Fc showed potent preventive and therapeutic effects. Compared to therapeutic effect of Fc-fusion proteins, SLAM-Nectin-Fc exhibited better efficacy in pre-entry treatment. So, receptor-targeted Fc-fusion proteins provided a new method for the control of canine distemper.

## Materials and methods

### Cells and virus

Human embryonic kidney (HEK) 293T cells were cultured in growth media composed of Dulbecco's Modified Eagle Medium (DMEM, Hyclone, United States) containing 10% fetal bovine serum (FBS) (BI, State of Israel), 100 U/mL penicillin and 0.1 mg/mL streptomycin at 37°C in 5% CO_2_. Vero cells stably expressing canine SLAM were maintained in above medium additionally added 0.5mg/mL G418. CDV SD16F strain was grown in above Vero cells.

### Plasmids construction

**Figure 1:**
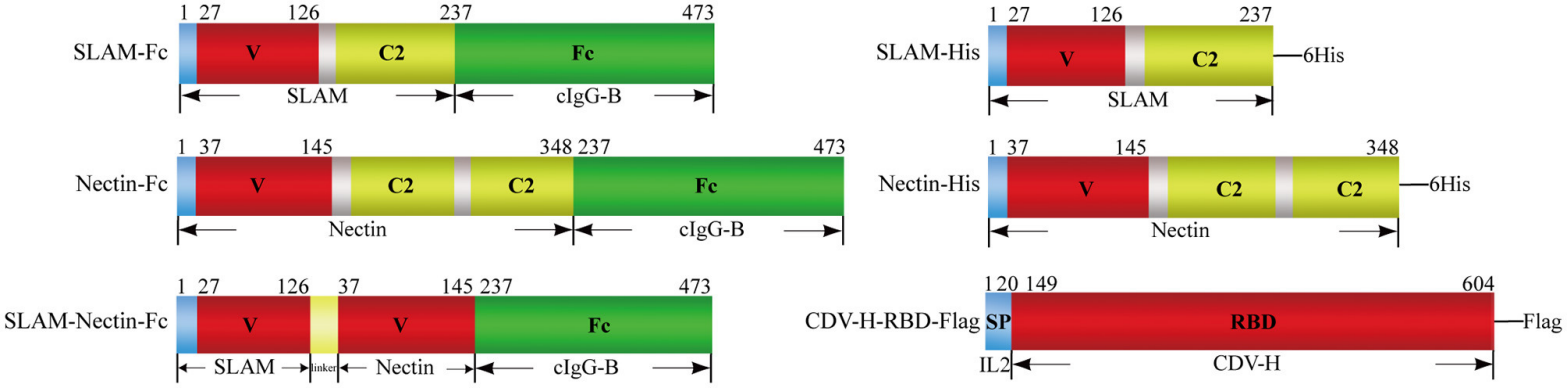
Schematic representation of proteins used in the present study. Receptor-Fc fusion proteins **(Left)** composed the extracellular portions of SLAM or/and Nectin-4 fused to the Fc portion of canine IgG-B(cIgG-B), the extracellular domain of SLAM or Nectin-4 was also tagged with 6×Histidine (6His) **(Right upper)**. V: V-set domain, C2: C2-set domain. CDV-H-RBD-Flag protein **(Right lower)** was designed by joining with receptor binding domain (RBD) of CDV-H protein and Flag-tag (DYKDDDDK) in the downstream of signal peptide from interleukin 2(IL2).

A total of six recombinant plasmids were designed in this experiment. For receptor-Fc proteins, extracellular domain of canine SLAM (GenBank Accession Number: NM_001003084.1) or Nectin-4 (GenBank Accession Number: NM_001313853.1) were designed to fuse with the Fc region of canine IgG-B (GenBank Accession Number: AF354265.1) respectively. Besides, a protein with combined V region of SLAM and Nectin-4 joined with the Fc region was also designed (Figure 1). At the same time, another three proteins were design for binding experiment. His-tag (HHHHHH) was used to construct SLAM-His and Nectin-His proteins, RBD of CDV-H protein from SD16F strain (GenBank Accession Number: MH337872.1) was joined with Flag-tag (DYKDDDDK) to construct CDV-H-RBD-Flag protein (Figure 1). All sequences of above constructs were ligated into eukaryotic expression vector pcDNA3.1 (+) at *Hin*d III and *Eco*R I restriction sites to construct the expression vectors pcDNA3.1-SLAM-Fc, pcDNA3.1-Nectin-Fc, pcDNA3.1-SLAM-Nectin-Fc, pcDNA3.1-SLAM-His, pcDNA3.1-Nectin-His and pcDNA3.1-CDV-H-RBD-Flag. All of coding sequences were codon-optimized and commercially synthesized by Sangon Biotech (Shanghai, China).

### Proteins transient expression

All of plasmids were transfected into HEK293T cells with PEI (Transporter 5 transfection reagent, Polysciences, United States) following the manufacturer's instructions. In 6-well plates, 2 μg of each plasmid were transfected with 4 μL PEI per well. The supernatant and cells were collected 96 h post transfection for identification by reduced sodium dodecyl sulfate polyacrylamide gel electrophoresis (SDS-PAGE) and western blot. Briefly, proteins were transferred to a nitrocellulose membrane and block the membrane using blocking buffer (Takara, Japan). For receptor-Fc proteins, the membrane was probed with Fc domain-specific antibody (AffiniPure Rabbit Anti-Dog IgG-Fc fragment Specific, Jackson ImmunoResearch, United States) followed by Peroxidase-conjugated AffiniPure Goat Anti-Rabbit IgG(H+L) (Jackson ImmunoResearch) with the dilution of 1:5,000 in 1x Tris buffered saline with 0.05% Tween 20(TBST). For His-tag and Flag-tag proteins, His-tag monoclonal antibody (Affinity, United States) and Flag-tag monoclonal antibody (Affinity, United States) were used as a primary antibody, followed by Peroxidase-conjugated AffiniPure Goat Anti-Mouse IgG(H+L) (Jackson ImmunoResearch) with the dilution of 1:5,000 in 1xTBST. The membrane was washed with 1xTBST and the signal was developed using an ECL reagent (Tanon, China). The proteins were subsequently expressed in cells grown in T25 flasks for purification, 14 μg of each plasmid and 16 μl PEI per flask were used for transfection.

### Proteins purification

Cells and cell culture supernatant were collected for protein purification. The cell culture supernatant was collected and filtered by 0.45 μm membrane filter (Millipore, United States), then concentrated by ultrafiltration centrifuge tube (Amicon Ultra-15 Centrifugal Filter Units, Millipore). Cells were centrifuged and collected followed by ultrasonication, then the supernatant obtained by recentrifuged was used for purification. Receptor-Fc proteins were purified by affinity chromatography using a 1 mL HiTrap rProtein A FF column (Cytiva, United States). The column was washed with sodium phosphate (pH 7.0) followed by 0.1 M glycine-HCl (pH 3.5) for receptor-Fc proteins elution. Eluate fractions were instantly neutralized using 1 M Tris-HCl (pH 9.0). SLAM-His and Nectin-His were purified by His-tagged protein purification kit (CWBio, China) and 500 mM Imidazole for elution. CDV-H-RBD-Flag was purified by Anti-DYKDDDDK Affinity Gel (Yeasen, China), the elution method was the same as receptor-Fc proteins. Then each protein was concentrated by ultrafiltration centrifuge tube and replaced the buffer with phosphate-buffered saline (PBS, pH 7.4). The purified proteins were confirmed by reduced SDS-PAGE and western blot.

### CDV-H-RBD binding assay

The binding activities of the purified receptor proteins to CDV-H-RBD were analyzed by enzyme linked immunosorbent assay (ELISA). 96-well plate was coated with 100 ng of HEK293T-produced CDV-H-RBD-Flag, and incubated overnight at 4°C. The wells were blocked by 5% bovine serum albumin (BSA) for 2 h at 37°C. The plate was washed three times with 1x phosphate-buffered saline containing 0.05% Tween 20(PBST), and incubated for 2 h at 37°C with serially diluted (0 μg/mL, 0.625 μg/mL, 1.25 μg/mL, 2.5 μg/mL, 5 μg/mL, 10 μg/mL, 20 μg/mL) proteins of SLAM-Fc, Nectin-Fc, SLAM-Nectin-Fc, SLAM-His, Nectin-His produced from HEK293T cells, PBS was used as negative controls. After washing, an anti-Fc domain or anti-6X His-tag antibody diluted in 1xPBS were added into the wells and incubated for 2 h at 37°C. After washing again, HRP-fusion secondary antibody was added to wells and incubated for 2 h at 37°C. For detection, Tetramethylbenzidine (TMB) as the substrate solution was added into the plate after thorough washing. The enzymatic reactions were stopped by adding 1M H_2_SO_4_. OD450 value was obtained using 96-well microplate reader.

### Receptor-Fc proteins and His-tagged receptors competitively bind to CDV-H-RBD

The competing effects of receptor-Fc proteins and His-tagged receptors were analyzed by ELISA. 96-well plate was coated with 100 ng of HEK293T-produced CDV-H-RBD-Flag and incubated overnight at 4°C. The wells were blocked using 5% BSA for 2 h at 37°C. The plate was washed three times with 1xPBST and incubated with serially diluted proteins of SLAM-Fc, Nectin-Fc, SLAM-Nectin-Fc produced from HEK293T cells, and PBS as negative control for 2 h at 37°C. After washing, SLAM-His was added to SLAM-Fc and SLAM-Nectin-Fc group, and Nectin-His was added to Nectin-Fc and SLAM-Nectin-Fc group. His-tag proteins were added an equal amount of 20 μg/mL and incubated for 2 h at 37°C. After washing, His-tag monoclonal antibody diluted in 1xPBS were added into the wells and incubated for 2 h at 37°C. After washing again, add HRP-fusion secondary antibody to wells and incubated for 2 h at 37°C. The detection method is the same as the binding experiment.

### *In vitro* antiviral assay: pre-treatment

For pre-entry treatment, 100 μL (2 × 10^5^) of cells were seeded in 96-well plates and incubated at 37°C in 5% CO_2_. For pre-entry treatment, serially diluted (0 μg/mL, 0.02 μg/mL, 0.2 μg/mL, 2 μg/mL, 20 μg/mL, 200 μg/mL) receptor-Fc proteins were mixed individually with CDV SD16F strain at a multiplicity of infection (MOI =0.5), then incubated at 37°C for 1 h before viral adsorption for another 1 h. The cells were washed twice with 1xPBS followed by the addition of DMEM with 2% FBS after which cells were maintained under standard conditions for an additional 48 h. PBS was used as negative control instead of virus.

After 48 h, total RNAs were extracted from cell culture supernatant using a TaKaRa MiniBEST Viral RNA/DNA Extraction Kit (TaKaRa), and cDNAs were obtained using HiScript II 1st Strand cDNA Synthesis Kit (Vazyme, China) according to the manufacturer's instructions. Real-time fluorescent quantitative PCR (qPCR) was conducted to amplify a 121 bp region of CDV N gene, each 25 μL PCR reaction contained 5 μM forward primer (5′-GAGCAAGTTTGGATTCTG-3′), 5 μM reverse primer (5′-GCATCATCAACTTCTATGTC-3′) and 10 μM probe primer (5′-(FAM)TCTCCAACCAGCCTAATTGTCCTT(EcLipse)-3′) (TaKaRa). In addition to the primers and probe, each 25 μL reaction contained 2 μL of cDNA template, 12.5 μL Premix Ex Taq (Probe qPCR) (TaKaRa) and 8.5 μL of RNase-free ddH_2_O. The qPCR underwent 95°C for 30 s, followed by 40 cycles of 95°C for 5 s, 55°C for 10 s and 72°C for 20 s. For quantification of CDV viral load, a standard curve was performed with a recombinant plasmid pMD18-T-N containing CDV-N gene.

The Vero cells were fixed with 4% paraformaldehyde (Solarbio, China) for 20 min and incubated with 0.2% Triton-X 100 (Solarbio) for 10 min, followed by blocking with 5% BSA for 1 h at room temperature. Then cells were incubated with the anti-CDV-N monoclonal antibody (LvDu, Binzhou, China) at 37°C for 2 h, followed by washing with PBS thrice. After incubating with fluorescein isothiocyanate (FITC) conjugated rabbit anti-mouse IgG (H+L) secondary antibody (Wanleibio, China), and staining with nuclear staining dye (DAPI, Solarbio) in dark, cells were observed under fluorescence microscope, multiple random visual fields were selected to quantify syncytia size and number.

### *In vitro* antiviral assay: post-treatment

Vero cells were cultured in the same way as pre-treatment experiment. For the post-treatment, CDV SD16F strain (MOI = 0.5) was adsorbed for 1 h at 37°C, after washing the cells with 1xPBS, DMEM with 2% FBS was added. Various concentrations (0 μg/mL, 0.02 μg/mL, 0.2 μg/mL, 2 μg/mL, 20 μg/mL, 200 μg/mL) of receptor-Fc proteins were directly added to the culture medium and cells were maintained at 37°C in a 5% CO_2_ incubator for 48h. PBS was also used as negative control instead of virus. The procedure of follow-up experiment is the same as that of pre-treatment.

### Statistical analysis

All experiments were assayed in triplicate and repeated at least twice. Statistical analysis was performed using GraphPad Prism software. A log (agonist) vs. normalized response-Variable slope test was used for EC_50_ determination. A log (inhibitor) vs. normalized response-Variable slope test was used for IC_50_ determination. All values are presented as the mean ± standard deviation (SD) of three replicates. Multiple comparisons following one-way ANOVA were performed for statistical analysis. *P*-values < 0.05 were statistically significant (^*^p < 0.05, ^**^p < 0.01, ^***^p < 0.001, ^****^p < 0.0001).

## Results

### Proteins purification

**Figure 2:**
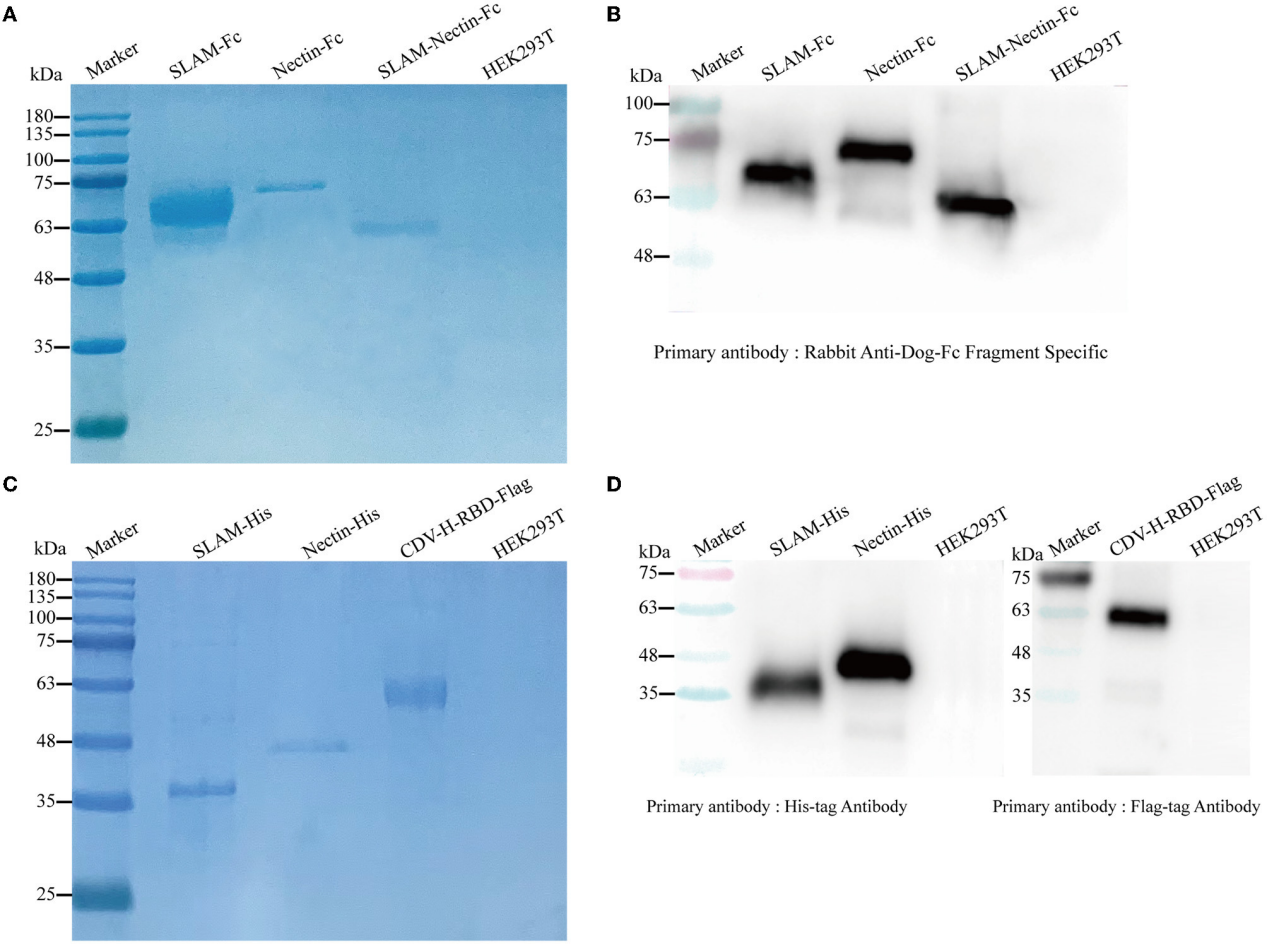
Detection of purified proteins by reduced SDS-PAGE **(A, C)** and Western blot **(B, D)**. **(A)** The culture supernatant was harvested and purified using HiTrap rProtein A FF column. The purified receptor-Fc fusion proteins were mixed with 5× loading buffer to conduct SDS-PAGE. HEK293T cells were used as negative control. **(B)** Rabbit Anti-Dog IgG-Fc fragment Specific antibody (1:200) was used as the primary antibody, and Peroxidase-conjugated Goat Anti-Rabbit antibody (1:5,000) was used as the secondary antibody. HEK293T cells were used as negative control. **(C)** The culture supernatant and cells were harvested, His-tagged proteins were purified using His-tagged protein purification kit, and CDV-H-RBD was purified using Anti-DYKDDDDK Affinity Gel. The purified recombinant proteins were mixed with 5× loading buffer to conduct SDS-PAGE. HEK293T cells were used as negative control. **(D)** His-tag monoclonal antibody (1:5,000) was used to verify His-tag proteins, Flag-tag monoclonal antibody (1:5,000) was used to verify CDV-H-RBD-Flag, and Peroxidase-conjugated Goat Anti-Mouse antibody (1:5,000) was used as the secondary antibody. HEK293T cells were used as negative control.

We generated Fc-fusion proteins by fusing the SLAM and Nectin-4 protein to the canine IgG-B Fc region. To produce the soluble proteins in HEK293T cells, the genes were incorporated into the pcDNA3.1 (+) eukaryotic system expression vector and subsequently transfection into HEK293T with PEI. To purify the proteins, we used rProtein A FF column, His-Tagged and Flag-Tagged purification Kit. We estimate that the proteins were relatively pure based on visual inspection of a Coomassie blue stained gel. Nectin-Fc, SLAM-Nectin-Fc, Nectin-His and CDV-H-RBD-Flag showed the protein band at 64 kDa, 54 kDa, 38 kDa and 54 kDa, respectively (Figures 2A, C), which was consistent with the predicted molecular weight of their monomers. Meanwhile, the protein bands of SLAM-Fc and SLAM-His were larger than the expected monomer sizes (53 kDa and 27 kDa) (Figures 2A, C), as previously reported (14). The specificity of HEK293T-produced proteins was confirmed by western bolt analysis using Fc-domain-specific, His-specific and Flag-specific antibody. The results indicated that each purified protein showed single band which the molecular weight was same as the profiles on Coomassie blue stained gel (Figures 2B, D).

### CDV-H-RBD binding assay

CDV invades and infects cells mainly by binding to SLAM and Nectin-4 receptor on host cells. The binding specificity of SLAM and Nectin to CDV-H-RBD was confirmed by ELISA. The purified CDV-H-RBD-Flag protein was immobilized in the 96-wells plate. Seven different dilutions of SLAM-His, Nectin-His, SLAM-Fc, Nectin-Fc, SLAM-Nectin-Fc, and PBS were added into the ELISA plate with triplicate wells.

**Figure 3:**
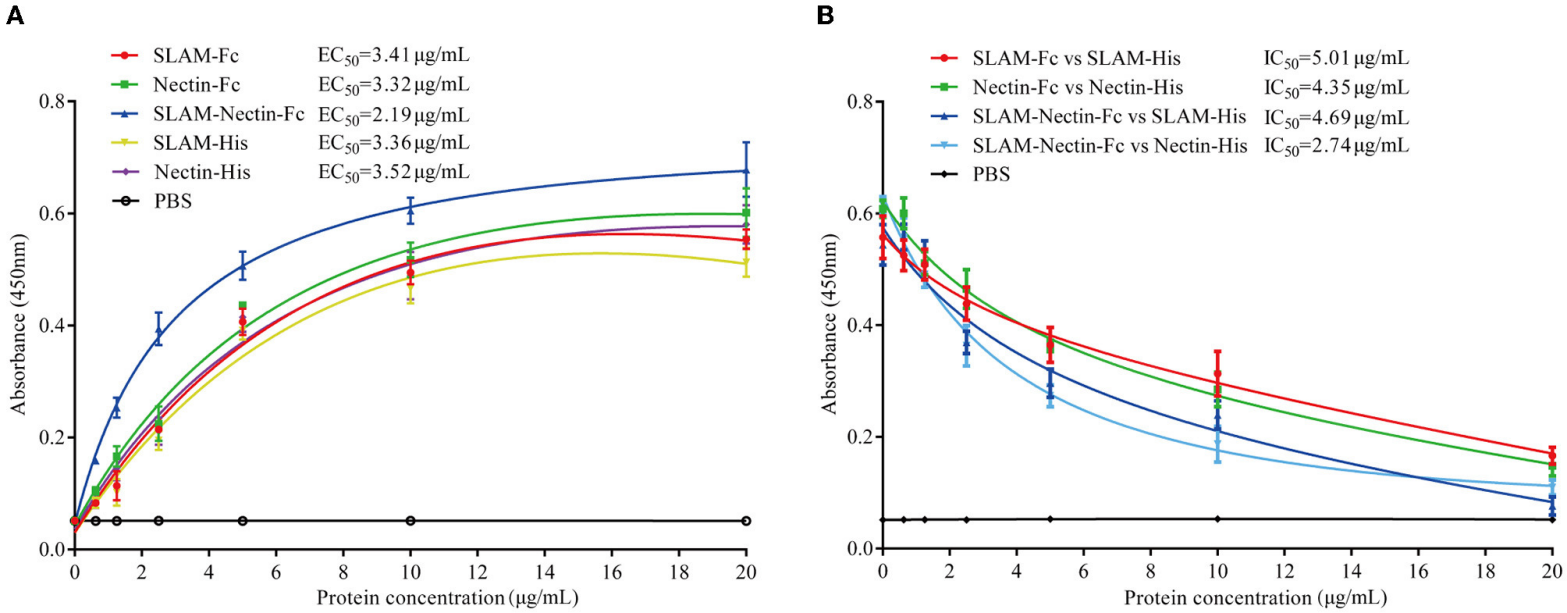
CDV-H-RBD binding and competition assay. **(A)** Binding activity of the purified receptor proteins with CDV-H-RBD-Flag was analyzed by ELISA. PBS buffer was used as negative control. Data are presented as mean ± SD of triplicates. **(B)** Competing effect of receptor-Fc fusion proteins and His-tagged receptor proteins to CDV-H-RBD-Flag was analyzed by ELISA. PBS buffer was used as negative control. Data are presented as mean ± SD of triplicates.

The results showed all of proteins produced substantially high OD signals with the CDV-H-RBD-Flag compared to the negative PBS (Figure 3A), indicating that both receptor-Fc proteins and His-tagged proteins efficiently bound to CDV-H-RBD. EC_50_ of receptor-Fc proteins (SLAM-Fc, Nectin-Fc, SLAM-Nectin-Fc) was 3.41 μg/mL, 3.32 μg/mL and 2.19 μg/mL, meanwhile, EC_50_ of SLAM-His and Nectin-His was 3.36 μg/mL and 3.52 μg/mL. Moreover, SLAM-Fc, Nectin-Fc, SLAM-Nectin-Fc, SLAM-His and Nectin-His showed extremely significant differences (*P* < 0.01) starting from 1.25 μg/mL, 1.25 μg/mL, 0.625 μg/mL, 2.5 μg/mL and 1.25 μg/mL, respectively.

In addition, the SLAM-Nectin-Fc group showed extremely significant differences (*P* < 0.01) compared with other proteins groups under 2.5 μg/mL. When the concentration varied at 5 μg/mL to 10 μg/mL, the SLAM-Nectin-Fc also showed extremely significant differences (*P* < 0.01) compared with other proteins except Nectin-Fc that showed significant differences (0.01 < *P* < 0.05). It indicated the binding effect of SLAM-Nectin-Fc to CDV-H-RBD was higher than other receptor proteins.

### Receptor-Fc proteins and His-tagged receptors competitively bind to CDV-H-RBD

The results showed that the higher the receptor-Fc protein concentration, the lower the OD signals (Figure 3B). IC_50_ of four groups (SLAM-Fc vs. SLAM-His, Nectin-Fc vs. Nectin-His, SLAM-Nectin-Fc vs. SLAM-His, SLAM-Nectin-Fc vs. Nectin-His) was 5.01 μg/mL, 4.35 μg/mL, 4.69 μg/mL and 2.74 μg/mL, respectively. Due to the better effect of the binding of SLAM-Nectin-Fc and CDV-H-RBD, SLAM-Nectin-Fc shows better competitive effect at the same concentration. When the concentration of receptor-Fc proteins was at 5 μg/mL, the group of SLAM-Nectin-Fc vs. Nectin-His showed extremely significant differences (*P* < 0.01) compared with the group of SLAM-Fc vs. Nectin-His. When the concentration of receptor-Fc proteins was at 20 μg/mL, the group of SLAM-Nectin-Fc vs. SLAM-His showed extremely significant differences (*P* < 0.01) compared with the group of SLAM-Fc vs. SLAM-His. These data suggested that receptor-Fc proteins can be used as a pseudo-receptor to bind the virus, then prevent the virus from binding to native receptor.

### *In vitro* antiviral assay: pre-treatment

**Figure 4:**
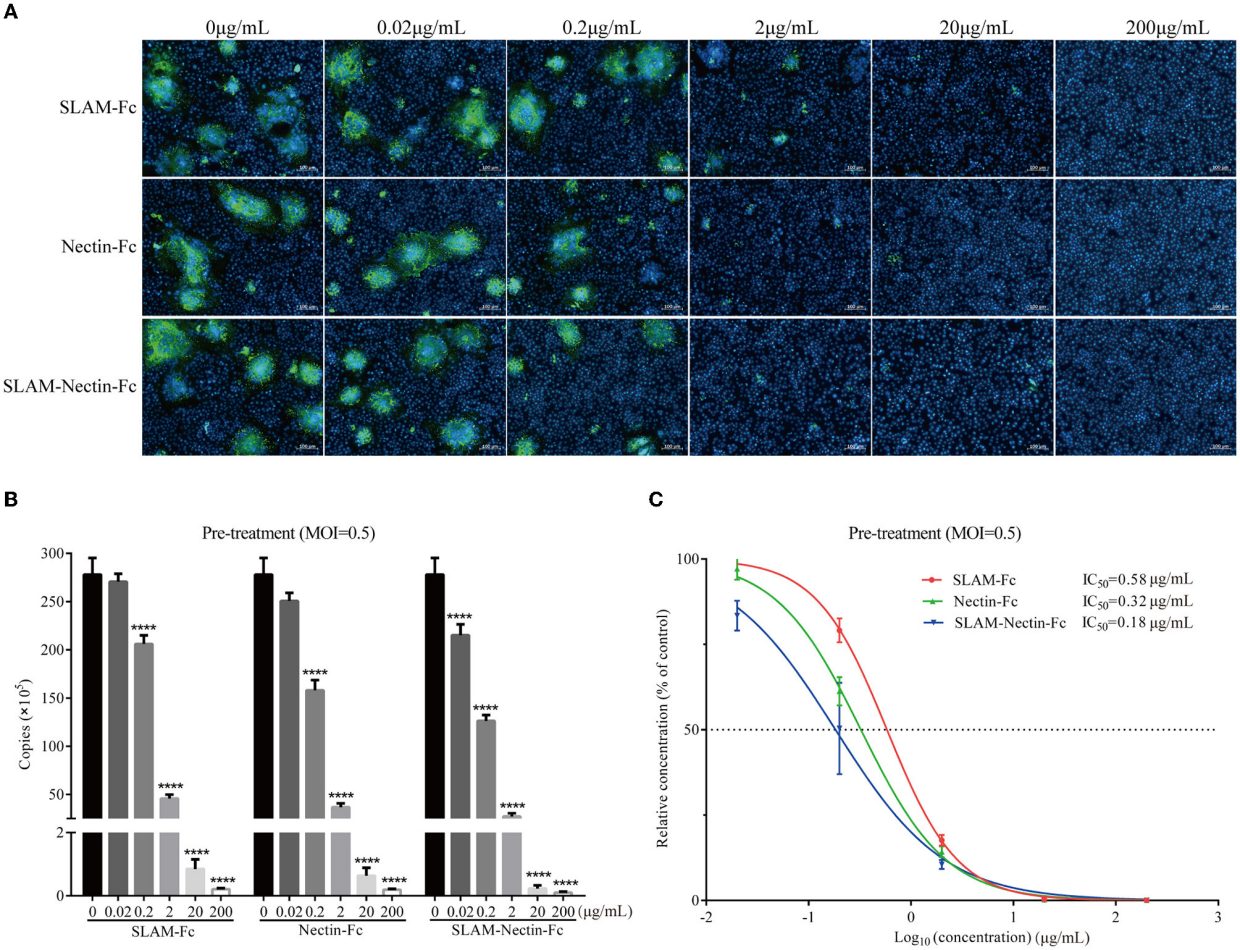
Dose-dependent effect of receptor-Fc fusion proteins on CDV inhibition at the pre-infection phase. **(A)** Indirect immunofluorescence assay revealed CDV infection profiles in Vero cells which were pre-treated with six concentrations of SLAM-Fc, Nectin-Fc, and SLAM-Nectin-Fc. **(B)** Viral genome copies of CDV in culture supernatant of Vero cells, which were pre-treated with six concentrations of SLAM-Fc, Nectin-Fc, and SLAM-Nectin-Fc. Data were analyzed by one-way ANOVA using GraphPad Prism software. Significance is presented as ****p < 0.0001. **(C)** Efficacy of receptor-Fc proteins for CDV inhibition in Vero cells, which were pre-treated by six concentrations of SLAM-Fc, Nectin-Fc, and SLAM-Nectin-Fc. The data were showed as mean ± SD of triplicates in individual concentrations.

We investigated whether receptor-Fc proteins can affect the cytopathic effect (CPE) induced by CDV, namely syncytia formation, through indirect immunofluorescence assay. The magnitude of fluorescence represents the size and number of syncytia. Strikingly, a clear concentration-dependent decrease in number and size of the syncytia was observed. When the protein concentration was 2 μg/mL, there was almost no fluorescence in three groups (Figure 4A).

As for viral RNA level, compared with 0 μg/mL group (virus control group), the viral genome copies of SLAM-Fc and Nectin-Fc group showed extremely significant differences (*P* < 0.01) starting at 0.2 μg/mL, but SLAM-Nectin-Fc showed extremely significant differences (*P* < 0.01) starting at 0.02 μg/mL. When the concentration of Fc-fusion proteins was at 0.2 μg/mL, the Nectin-Fc group had significantly lower viral genome copies than SLAM-Fc group (*P* < 0.01), while the viral genome copies of SLAM-Nectin-Fc group were significantly lower than that of SLAM-Fc (*P* < 0.01) and Nectin-Fc (*P* < 0.05) group (Figure 4B). In addition, the 50% inhibition concentration (IC_50_) of SLAM-Fc, Nectin-Fc, and SLAM-Nectin-Fc group was 0.58 μg/mL, 0.32 μg/mL and 0.18 μg/mL, respectively (Figure 4C). Among three groups, SLAM-Nectin-Fc emerged as the most potent inhibitors of CDV infection.

### *In vitro* antiviral assay: post-treatment

**Figure 5:**
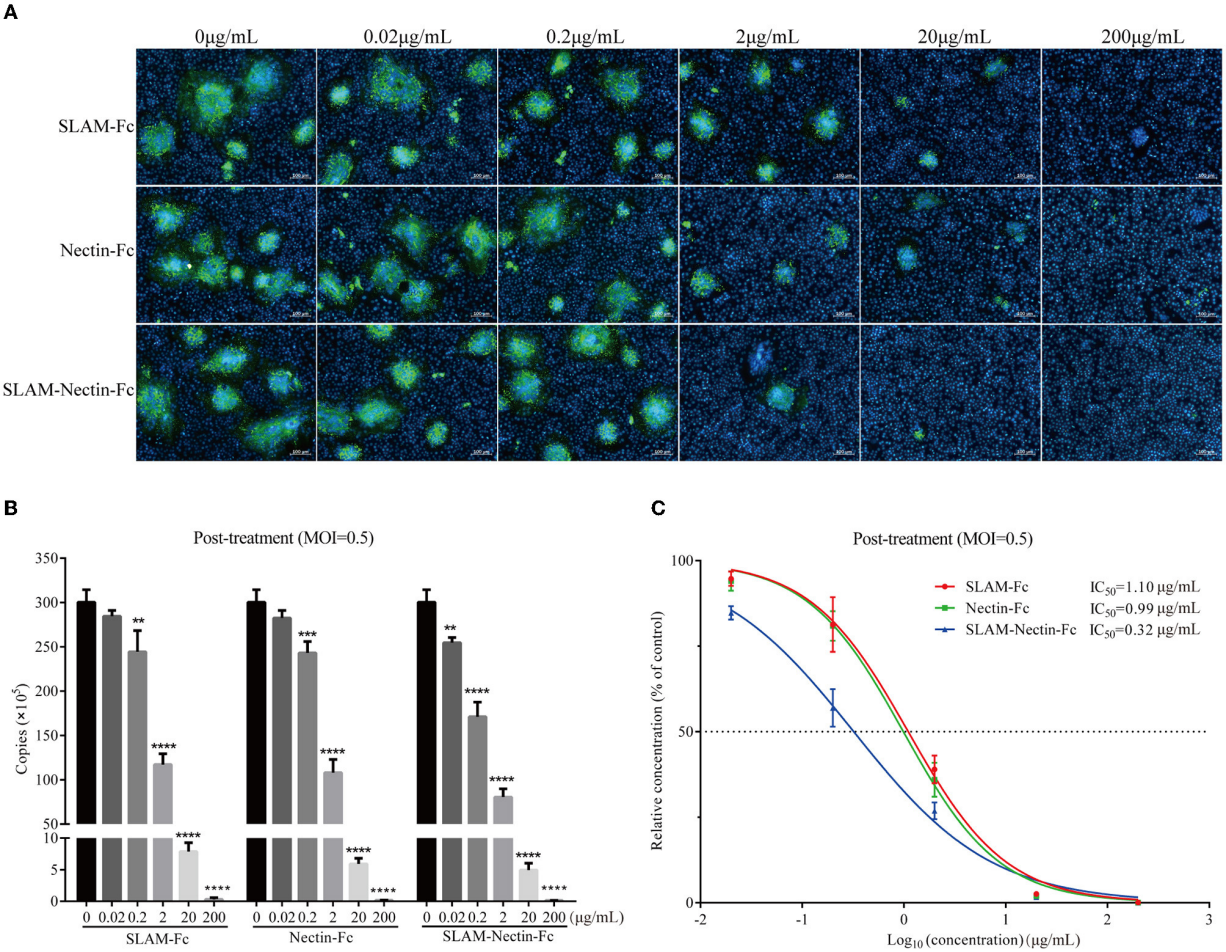
Dose-dependent effect of receptor-Fc proteins on CDV inhibition at the post-infection phase. **(A)** Indirect immunofluorescence assay revealed CDV infection profiles in Vero cells which were post-treated with six concentrations of SLAM-Fc, Nectin-Fc, and SLAM-Nectin-Fc. **(B)** Viral genome copies of CDV in culture supernatant of Vero cells, which were post-treated with six concentrations of SLAM-Fc, Nectin-Fc, and SLAM-Nectin-Fc. Data were analyzed by one-way ANOVA using GraphPad Prism software. Significance is presented as **p < 0.01, ***p < 0.001, ****p < 0.0001. **(C)** Efficacy of receptor-Fc proteins for CDV inhibition in Vero cells, which were post-treated by six concentrations of SLAM-Fc, Nectin-Fc, and SLAM-Nectin-Fc. The data were showed as mean ± SD of triplicates in individual concentrations.

Next, we tested the efficacy of the post-infection treatment. According to the result of indirect immunofluorescence assay, it was still observed that the number and size of syncytia decreased with increasing receptor-Fc proteins concentration. Fluorescence was significantly decreased at concentration 2 μg/mL, and no fluorescence was observed in three groups when the protein concentration was 200 μg/mL. Based on the result of 20 μg/mL, SLAM-Nectin-Fc also emerged as the most potent inhibitors of CDV infection compared with SLAM-Fc and Nectin-Fc (Figure 5A).

For viral RNA synthesis, compared with 0 μg/mL group, the viral genome copies of SLAM-Fc and Nectin-Fc group also showed extremely significant differences (*P* < 0.01) starting at 0.2 μg/mL, SLAM-Nectin-Fc showed extremely significant differences (*P* < 0.01) starting at 0.02 μg/mL (Figure 5B), this showed that the minimum effective concentration (MEC) of three receptor-Fc proteins was 0.2 μg/mL, 0.2 μg/mL and 0.02 μg/mL, respectively, the same as pre-treatment assay. When the concentration of Fc-fusion proteins was at 0.2 μg/mL, there was no significant difference in viral load between SLAM-Fc group and Nectin-Fc group (*P* > 0.05), while the group of SLAM-Nectin-Fc have significantly lower viral genome copies than the other two Fc–fusion protein groups (*P* < 0.01). The IC_50_ of SLAM-Fc, Nectin-Fc, and SLAM-Nectin-Fc group was 1.10 μg/mL, 0.99 μg/mL and 0.32 μg/mL (Figure 5C), respectively.

## Discussion

Infection caused by CDV is a highly contagious disease with high incidence and fatality in the multi-hosts including canine population. Control of CDV infection in canine population is highly important since canine animals have historically been considered the major reservoir for CDV, spill over from viral reservoirs in canines has implications for endangered wildlife and other hosts (7, 8). It is of significance to develop novel effective and safe antiviral agents against CDV, since currently used vaccines have the drawbacks of immunization failures or vaccine-induced CD cases (29). In this study, we expressed three soluble receptor-Fc fusion proteins (SLAM-Fc, Nectin-Fc, and SLAM-Nectin-Fc) in HEK293T cells, and confirmed the receptor-Fc fusion proteins had potent antiviral activity against CDV *in vitro*.

Previous antiviral studies for CDV have achieved great progress, mainly focused on natural compounds and synthetic inhibitors. Natural compounds such as South African plants (30), flavonoids and phenolic acids (31), showed the ability to inhibit CDV *in vitro*. Synthetic substances, for example, ribavirin (RBV) and favipiravir were purine analog drug with a primary antiviral mechanism of suppressing the RNA-dependent RNA polymerase (RdRP) activity, also exhibited effective suppression of CDV replication *in vitro* (32, 33). Short interfering RNA and A77 1726 suppressed CDV replication by inhibiting viral nucleotide synthesis (34, 35). Other antiviral inhibitors including polyclonal antibody showed potential therapeutic effects against CDV infection in dogs (36). However, chemical drugs may lead to generation of drug-resistant mutants, xenogeneic polyclonal or monoclonal antibody has disadvantages of heterologous reactions and cross-protection, which restricted its clinical application, therefore, no antiviral molecular was currently approved for CDV control. Here, we provide a new biological antiviral strategy by targeting to receptor proteins instead of high varied CDV, which greatly decreases the possibility of escaping by CDV variants.

SLAM and Nectin-4 are CDV functional receptors expressed by host immune cells and epithelial cells respectively (10). Blocking the binding of CDV-H-RBD and functional receptors (SLAM and Nectin-4) at the early stage of virus infection will be an effective approach. Recombinant receptor-Fc proteins generated by fusing SLAM or/and Nectin-4 extracellular domain to the N terminal of canine IgG-B Fc region may be a promising choice for early CD treatment, because the presence of the Fc domain may confer greater stability to some proteins, and enhance the expression of proteins in mammalian cells that are otherwise difficult to produce (37). Moreover, Fc domain increases their half-life and maintains a proper blood concentration for long-term therapeutic effects (38–40). Fc region from canine IgG-B increases the likelihood that the recombinant receptor proteins will be both active and recognized as self by the recipient species, thereby overcoming immunogenicity concerns. Here, we show that the recombinant Fc-fusion receptors have robust competitive binding affinity for CDV-H-RBD, and potent prophylactic and therapeutic efficacy against CDV infection *in vitro*, it indicated the caninised receptor-Fc proteins may be used as decoy antibody to block viral entry and prevent CDV infections.

Based on the results of pre-entry assay and post-treatment assay, receptor-Fc fusion proteins had neutralizing activities against CDV *in vitro*, and exhibited better inhibition effect in pre-entry assay than post-treatment assay. This is because virus-receptor binding is the first step in viral replication, and receptor-Fc fusion proteins acting as receptor blockers inhibit the early stages of viral replication. Once the virus has already entered and infected cells, inhibiting effect will be compromised. On the contrary, favipiravir is more effective post CDV exposure, since it must have enough time to enter cells and switch to active form before its antiviral effects exhibit (33). Meanwhile, SLAM-Nectin-Fc showed better anti-virus effect than SLAM-Fc and Nectin-Fc. These data indicates that receptor-Fc proteins, especially SLAM-Nectin-Fc, have the potential to be developed as a preventive inhibitor of CDV. Once the antiviral activity of receptor-Fc proteins against CDV *in vivo* was further determined, we can clinically use SLAM-Nectin-Fc solely or combination of SLAM-Fc and Nectin-Fc to confer a synergetic biological effect, as previously reported (41). Further analysis will determine whether those promising candidates can be considered in the treatment of CDV as well as for the development of antiviral drugs against other viruses of the genus *Morbillivirus*, since they have similar virus-receptor interaction (42).

In conclusion, we showed that HEK293T cells can rapidly and effectively produce functional receptor-Fc fusion proteins SLAM-Fc, Nectin-Fc, and SLAM-Nectin-Fc. More importantly, we have confirmed that SLAM-Fc, Nectin-Fc, and SLAM-Nectin-Fc have the potential to be developed as anti-CDV agents, and the efficacy of SLAM-Nectin-Fc was better than SLAM-Fc and Nectin-Fc. However, animal studies are needed to confirm the efficacy and safety of receptor-Fc proteins against CDV before the goal of treatment or prevention can be further achieved.

## Data availability statement

The original contributions presented in the study are included in the article/supplementary material, further inquiries can be directed to the corresponding author/s.

## Author contributions

LS performed the experiments and prepared original draft. HS participated in data analysis. JH conceived, designed and supervised the research project, including reviewing the data, and revising the manuscript. All authors contributed to the discussion of data analysis and manuscript writing, read and approved the final manuscript.

## References

[1] Wilkes RP. Canine distemper virus in endangered species: species jump, clinical variations, and vaccination. Pathogens. (2023) 12:57. 10.3390/pathogens12010057 36678405 PMC9862170

[2] Alfano F Lanave G Lucibelli MG Miletti G D'Alessio N Gallo A. Canine distemper virus in autochtonous and imported dogs, Southern Italy (2014-2021). Animals. (2022) 12:2852. 10.3390/ani12202852 36290237 PMC9597831

[3] Kličková E Cerníková L Dumondin A Bártová E Budíková M Sedlák K. Canine distemper virus in wild carnivore populations from the Czech Republic (2012-2020) : occurrence, geographical distribution, and phylogenetic analysis. Life. (2022) 12:289. 10.3390/life12020289 35207575 PMC8874654

[4] Rohowsky-Kochan C Davidow A Dowling P Cook SD. Increased frequency of canine distemper virus-specific antibodies in multiple sclerosis. Brain Behav. (2021) 11:e01920. 10.1002/brb3.1920 33300690 PMC7821626

[5] Batista Linhares M Whiteley HE Samuelson JP Hsiao SH Stern AW Sprandel IT. Sylvatic canine morbillivirus in captive panthera highlights viral promiscuity and the need for better prevention strategies. Pathogens. (2021) 10:544. 10.3390/pathogens10050544 33946447 PMC8147164

[6] Feijóo G Yamasaki K Delucchi L Verdes JM. Central nervous system lesions caused by canine distemper virus in 4 vaccinated dogs. J Vet Diagn Invest. (2021) 33:640–7. 10.1177/10406387211009210 33870768 PMC8229831

[7] Liu Y Liu C Ding H Cao Y Sun Z Wu H. A highly virulent canine distemper virus strain isolated from vaccinated mink in China. Virus Genes. (2021) 57:266–75. 10.1007/s11262-021-01837-w 33950332

[8] Kennedy JM Earle JAP Omar S Abdullah H Nielsen O Roelke-Parker ME. Canine and phocine distemper viruses: global spread and genetic basis of jumping species barriers. Viruses. (2019) 11:944. 10.3390/v11100944 31615092 PMC6833027

[9] Takeda M Seki F Yamamoto Y Nao N Tokiwa H. Animal morbilliviruses and their cross-species transmission potential. Curr Opin Virol. (2020) 41:38–45. 10.1016/j.coviro.2020.03.005 32344228

[10] Zhao J Ren Y. Multiple receptors involved in invasion and neuropathogenicity of canine distemper virus: a review. Viruses. (2022) 14:1520. 10.3390/v14071520 35891500 PMC9317347

[11] Gast M Kadzioch NP Milius D Origgi F Plattet P. Oligomerization and cell egress controlled by two microdomains of canine distemper virus matrix protein. mSphere. (2021) 6:e01024–20. 10.1128/mSphere.01024-20 33853875 PMC8546710

[12] Herren M Shrestha N Wyss M Zurbriggen A Plattet P. Regulatory role of the morbillivirus attachment protein head-to-stalk linker module in membrane fusion triggering. J Virol. (2018) 92:e00679–18. 10.1128/JVI.00679-18 29997204 PMC6146710

[13] Fukuhara H Ito Y Sako M Kajikawa M Yoshida K Seki F. Specificity of morbillivirus hemagglutinins to recognize SLAM of different species. Viruses. (2019) 11:761. 10.3390/v11080761 31430904 PMC6722581

[14] Kalbermatter D Jeckelmann JM Wyss M Shrestha N Pliatsika D Riedl R. Structure and supramolecular organization of the canine distemper virus attachment glycoprotein. Proc Natl Acad Sci U S A. (2023) 120:e2208866120. 10.1073/pnas.2208866120 36716368 PMC9963377

[15] Wang R Wang X Zhai J Zhang P Irwin DM Shen X. A new canine distemper virus lineage identified from red pandas in China. Transbound Emerg Dis. (2022) 69:e944–52. 10.1111/tbed.14370 34724331

[16] Sawatsky B Cattaneo R Von Messling V. Canine distemper virus spread and transmission to naive ferrets: selective pressure on signaling lymphocyte activation molecule-dependent entry. J Virol. (2018) 92:e00669–18. 10.1128/JVI.00669-18 29793948 PMC6052283

[17] Wang Y Chen J Hu B Gong C Shi N Liu M. Mink SLAM V-region V74I substitutions contribute to the formation of syncytia induced by canine distemper virus. Front Vet Sci. (2021) 7:570283. 10.3389/fvets.2020.570283 33585591 PMC7874165

[18] Pratakpiriya W Seki F Otsuki N Sakai K Fukuhara H Katamoto H. Nectin4 is an epithelial cell receptor for canine distemper virus and involved in neurovirulence. J Virol. (2012) 86:10207–10. 10.1128/JVI.00824-12 22761370 PMC3446623

[19] Delpeut S Noyce RS Richardson CD. The V domain of dog PVRL4 (nectin-4) mediates canine distemper virus entry and virus cell-to-cell spread. Virology. (2014) 454–5:109–17. 10.1016/j.virol.2014.02.014 24725937

[20] Gradauskaite V Khosravi M Plattet P. Selective SLAM/CD150 receptor-detargeting of canine distemper virus. Virus Res. (2022) 318:198841. 10.1016/j.virusres.2022.198841 35649483

[21] Uzawa A Yamashita J Kuwabara S. Fc fusion protein as a novel treatment for Myasthenia Gravis. Brain Nerve. (2019) 71:525–30. 10.11477/mf.1416201306 31089000

[22] Alleva DG Delpero AR Scully MM Murikipudi S Ragupathy R Greaves EK. Development of an IgG-Fc fusion COVID-19 subunit vaccine, AKS-452. Vaccine. (2021) 39:6601–13. 10.1016/j.vaccine.2021.09.077 34642088 PMC8491978

[23] Huang Y Lin S Zhan F Xiao L Zhan Y Wang R. OX40-Fc fusion protein alleviates PD-1-Fc-aggravated rheumatoid arthritis by inhibiting inflammatory response. Comput Math Methods Med. (2022) 2022:6244175. 10.1155/2022/6244175 35222687 PMC8872694

[24] Rattanapisit K Srifa S Kaewpungsup P Pavasant P Phoolcharoen W. Plant-produced recombinant Osteopontin-Fc fusion protein enhanced osteogenesis. Biotechnol Rep. (2019) 21:e00312. 10.1016/j.btre.2019.e00312 30847284 PMC6389792

[25] LiuPWysockiJSoumaTYeMRamirezVZhouB. Novel ACE2-Fc chimeric fusion provides long-lasting hypertension control and organ protection in mouse models of systemic renin angiotensin system activation. Kidney Int. (2018) 94:114–25. 10.1016/j.kint.2018.01.02929691064

[26] Castilho A Schwestka J Kienzl NF Vavra U Grünwald-Gruber C Izadi S. Generation of enzymatically competent SARS-CoV-2 decoy receptor ACE2-Fc in glycoengineered Nicotiana benthamiana. Biotechnol J. (2021) 16:e2000566. 10.1002/biot.202000566 33481336 PMC7995010

[27] Mou H Quinlan BD Peng H Liu G Guo Y Peng S. Mutations derived from horseshoe bat ACE2 orthologs enhance ACE2-Fc neutralization of SARS-CoV-2. PLoS Pathog. (2021) 17:e1009501. 10.1371/journal.ppat.1009501 33836016 PMC8059821

[28] Siriwattananon K Manopwisedjaroen S Kanjanasirirat P Budi Purwono P Rattanapisit K Shanmugaraj B. Development of plant-produced recombinant ACE2-Fc fusion protein as a potential therapeutic agent against SARS-CoV-2. Front Plant Sci. (2021) 11:604663. 10.3389/fpls.2020.604663 33584747 PMC7874119

[29] Tamukai K Minami S Kurihara R Shimoda H Mitsui I Maeda K. Molecular evidence for vaccine-induced canine distemper virus and canine adenovirus 2 coinfection in a fennec fox. J Vet Diagn Invest. (2020) 32:598–603. 10.1177/1040638720934809 32560597 PMC7438639

[30] Bagla VP McGaw LJ Eloff JN. The antiviral activity of six South African plants traditionally used against infections in ethnoveterinary medicine. Vet Microbiol. (2012) 155:198–206. 10.1016/j.vetmic.2011.09.015 21982126

[31] Carvalho OV Botelho CV Ferreira CG Ferreira HC Santos MR Diaz MA. In vitro inhibition of canine distemper virus by flavonoids and phenolic acids: implications of structural differences for antiviral design. Res Vet Sci. (2013) 95:717–24. 10.1016/j.rvsc.2013.04.013 23664014

[32] Lanave G Cavalli A Martella V Fontana T Losappio R Tempesta M. Ribavirin and boceprevir are able to reduce Canine distemper virus growth in vitro. J Virol Methods. (2017) 248:207–11. 10.1016/j.jviromet.2017.07.012 28760649

[33] Xue X Zhu Y Yan L Wong G Sun P Zheng X. Antiviral efficacy of favipiravir against canine distemper virus infection in vitro. BMC Vet Res. (2019) 15:316. 10.1186/s12917-019-2057-8 31477101 PMC6720089

[34] de Carvalho OV Rebouças Santos M Lopes Rangel Fietto J Pires Moraes M de Almeida MR Costa Bressan G. Multi-targeted gene silencing strategies inhibit replication of canine morbillivirus. BMC Vet Res. (2020) 16:448. 10.1186/s12917-020-02671-2 33213424 PMC7676405

[35] Li Y Yi L Cheng S Wang Y Wang J Sun J. Inhibition of canine distemper virus replication by blocking pyrimidine nucleotide synthesis with A77 1726, the active metabolite of the anti-inflammatory drug leflunomide. J Gen Virol. (2021) 102:10.1099/jgv.0.001534. 10.1099/jgv.0.001534 33416466

[36] Zhang J Cui D Zuo Y Zheng Z Wu F Li W. Donkey-derived anti-CDV IgG, as a passive immunotherapy agent, can effectively increase survival rates of the experimental CDV-infected dogs. BMC Vet Res. (2021) 17:266. 10.1186/s12917-021-02982-y 34362358 PMC8344326

[37] Zhang J Carter J Siu S O'Neill JW Gates AH Delaney J. Fusion partners as a tool for the expression of difficult proteins in mammalian cells. Curr Pharm Biotechnol. (2010) 11:241–5. 10.2174/138920110791111898 20210749

[38] Ren W Sun H Gao GF Chen J Sun S Zhao R. Recombinant SARS-CoV-2 spike S1-Fc fusion protein induced high levels of neutralizing responses in nonhuman primates. Vaccine. (2020) 38:5653–8. 10.1016/j.vaccine.2020.06.066 32651113 PMC7311893

[39] Wang Y. Recombinant Elabela-Fc fusion protein has extended plasma half-life and mitigates post-infarct heart dysfunction in rats. Int J Cardiol. (2020) 300:217–8. 10.1016/j.ijcard.2019.06.043 31399296

[40] Niu YX Xu ZX Yu LF Lu YP Wang Y Wu C. Advances of research of Fc-fusion protein that activate NK cells for tumor immunotherapy. Int Immunopharmacol. (2022) 109:108783. 10.1016/j.intimp.2022.108783 35561479

[41] Wang H Li Y Shi G Wang Y Lin Y Wang Q. A novel antitumor strategy: simultaneously inhibiting angiogenesis and complement by targeting VEGFA/PIGF and C3b/C4b. Mol Ther Oncolytics. (2019) 16:20–9. 10.1016/j.omto.2019.12.004 31909182 PMC6940616

[42] Seki F Takeda M. Novel and classical morbilliviruses: current knowledge of three divergent morbillivirus groups. Microbiol Immunol. (2022) 66:552–63. 10.1111/1348-0421.13030 36151905

